# Supplementary material for: Change in brain asymmetry reflects level of acute alcohol intoxication and impacts on inhibitory control
Source: Sci Rep. 2023 Jun 24;13:10278. doi: 10.1038/s41598-023-37305-8 (PMC10290692; doi:10.1038/s41598-023-37305-8)
Supplement: Supplementary file 1 — Supplementary Information. [file 41598_2023_37305_MOESM1_ESM.pdf]

Supplementary Material

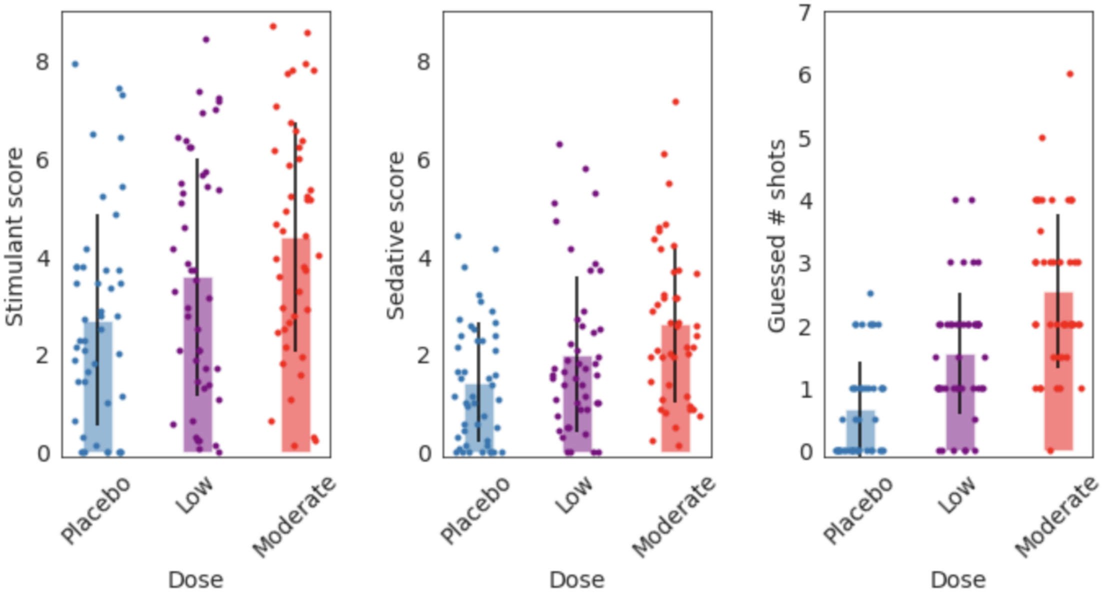

Supplementary Figure 1: Subjective measures of intoxication.

Shown are the subjective measures of intoxication for each participant (each dot) at different doses. For each measure, there was a significant difference between all dose pairs.

Stimulant scores (left) - paired t-test p-values: placebo/low = 9.21×10^{-3}, placebo/moderate = 1.49×10^{-5} , low/moderate = 1.50×10^{-3} ;

Sedative score (middle) - paired t-test p-values: placebo/low = 0.019, placebo/moderate = 1.32×10^{-5}, low/moderate = 9.98×10^{-3};

Guessed number of shots (right): paired t-test p-values: placebo/low = 3.31×10^{-5}, placebo/moderate = 2.60×10^{-11}, low/moderate = 5.18×10^{-7}

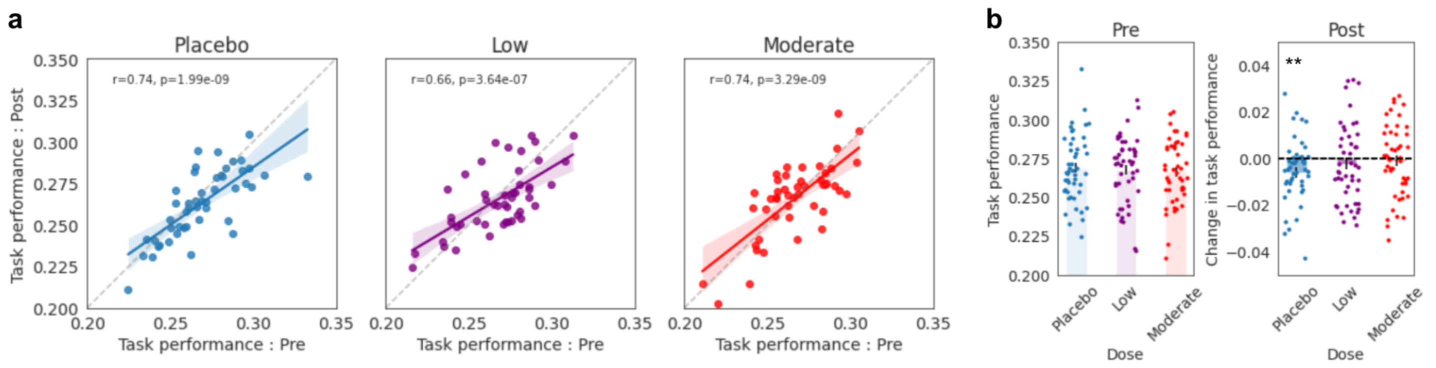

Supplementary Figure 2: Main effect of alcohol level on task performance as measured by reaction times.

a. Pre Go/No-go performance (reaction time measured in seconds) is plotted against post Go/No-go performance (reaction time measured in seconds) for placebo (left, blue), low (middle, purple), and moderate (right, red) levels of intoxication. Solid lines depict the line-of-best-fit and the shaded regions show the 95% confidence intervals. Dashed lines indicate the line of unity. Task performance is computed as the reaction times in the go-no-go blocks. **b.** Left: Colored bars show the average reaction times across the pre tasks for placebo, low, and moderate sessions separately. Right: The change in performance, measured as post reaction time - pre reaction time are shown for the different doses. Colored dots show individual subject measures (mean±s.e.m). Reaction times were significantly reduced in the post task compared to the pre task only for the placebo dose (paired t-test $p=9.48\times10^{-3}$).

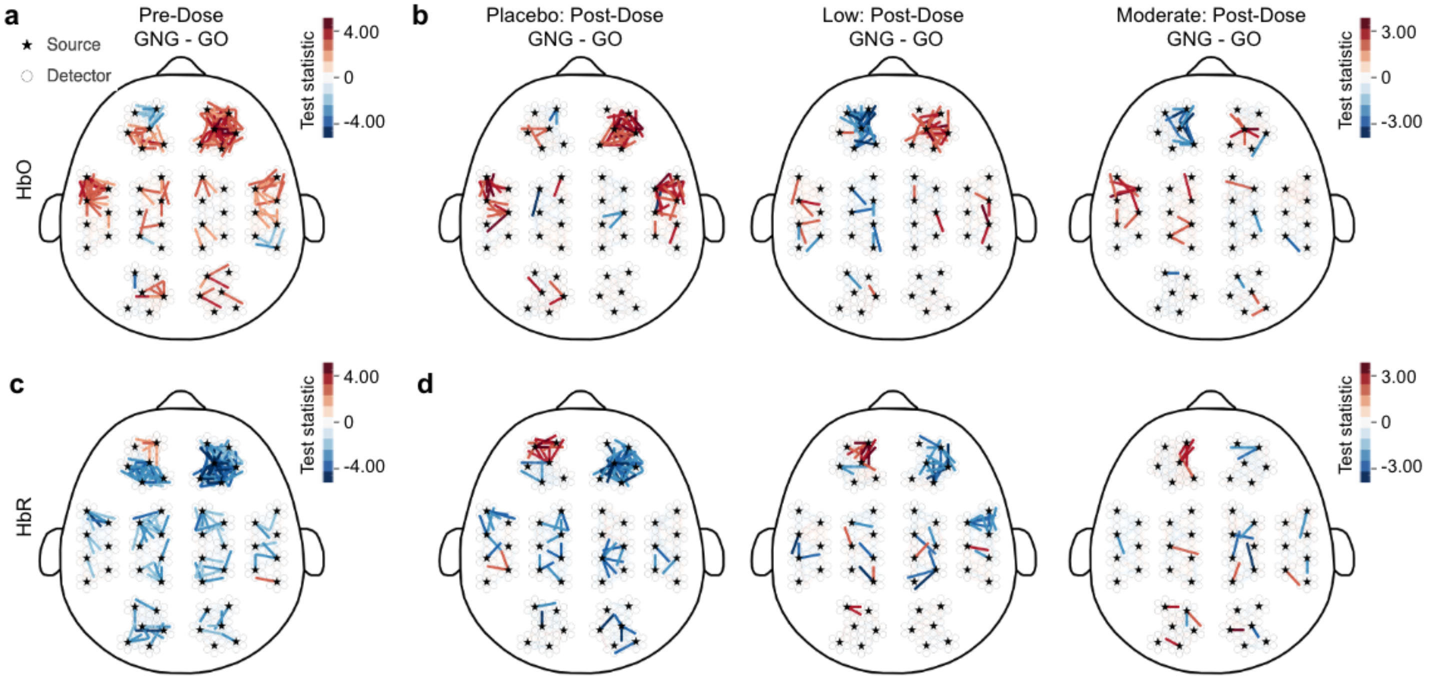

Supplementary Figure 3: Whole-head brain activation during an inhibitory control task under the influence of different alcohol doses as quantified by generalized linear models.

a-b. The contrast between brain activity (measured via HbO chromophore) during go-no-go blocks compared to the go-only blocks in pre-dose, and post-placebo, post-low, and post-moderate sessions (increased activation is indicated by warmer colors and decreased activation is indicated by cooler colors). Each line is an individual channel and only significant channels at $p<0.05$ are displayed. **c-d.** Same as (a-b) but for HbR. In all subpanels, the color scale indicates the t-statistic.

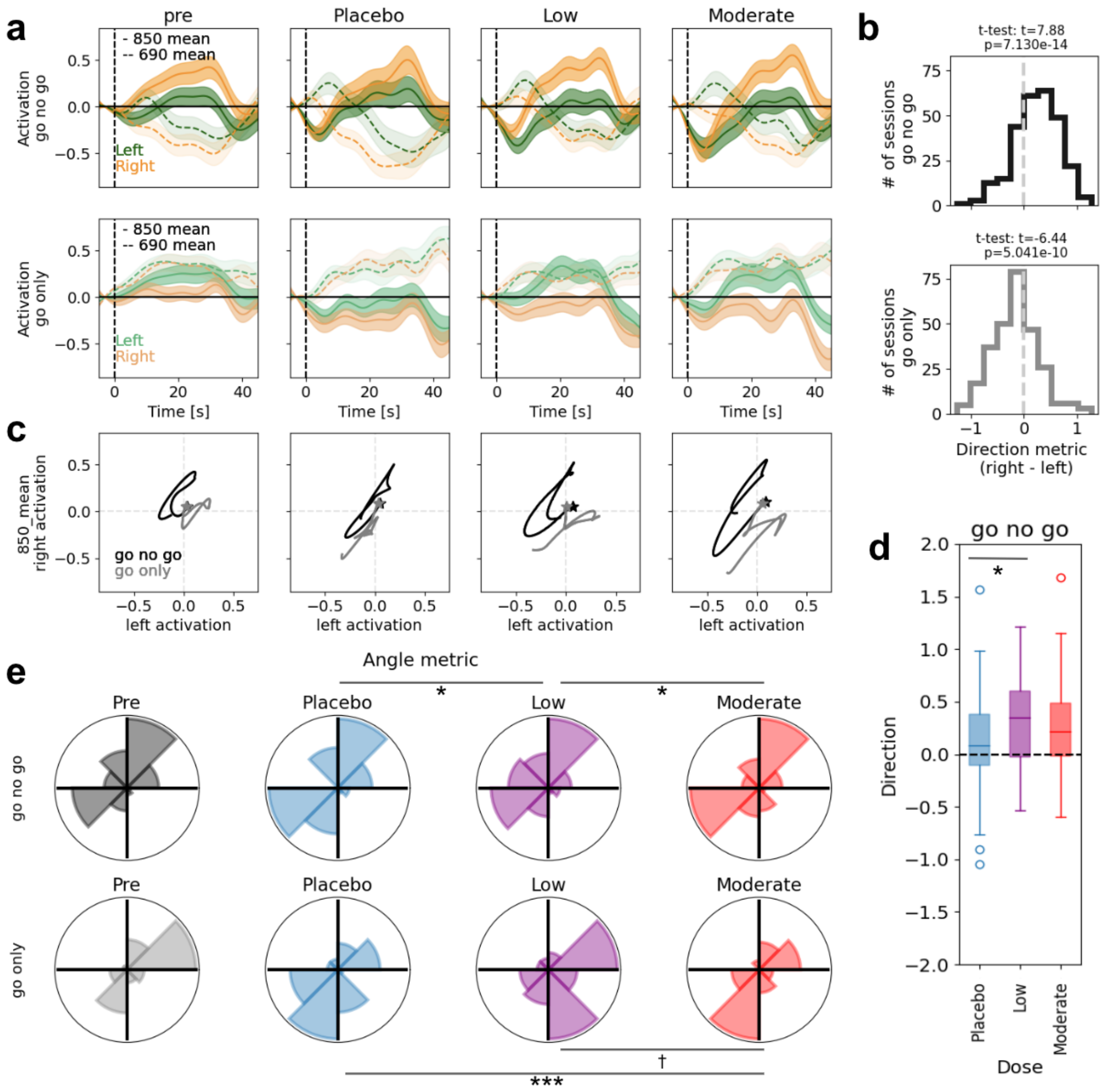

Supplementary Figure 4: DToF mean moment of 850nm shows altered patterns of prefrontal lateralization with different alcohol doses.

a. Block averaged time course of left (green) and right (orange) prefrontal activity (-mean moments) for go-no-go blocks (top) and go-only blocks (bottom) separated by session. From left to right, pre-dose and placebo-, low-, and moderate-dose time courses are shown (mean±s.e.m). Solid and dashed lines represent the 850nm and 690nm wavelengths mean moment respectively. Note the opposite trends in the two wavelengths, as expected. **b.** Population distribution of lateralization (direction metric) for go-no-go (top; black) and go-only (bottom; gray) blocks across all participants and sessions. Note the right lateralization for go-no-go blocks (shift towards positive values, t-test: t = 7.88, $p=7.13 \times 10^{-14}$) and left lateralization for go-only blocks (shift towards negative values, t-test: t=-6.44, $p=5.04 \times 10^{-10}$). **c.** Population average parametric curves showing the trajectory of right prefrontal vs. left prefrontal activity through time for go-only (gray) and go-no-go (black) blocks, split by pre-dose and session type, as in (a). Stars indicate initial time points. **d.** The distributions of the direction metric for go-no-go blocks across post-dose sessions (* significant difference between placebo and low dose: $p<0.05$). **e.** The distributions of the angle metric, split by pre-dose and session type, as in (a). * and *** indicate significant deviation from uniformity (at $p < 0.05$ and $p < 0.001$ respectively; rayleigh test; † trend towards significant) when computing pairwise difference in the angle metric between dosing sessions.

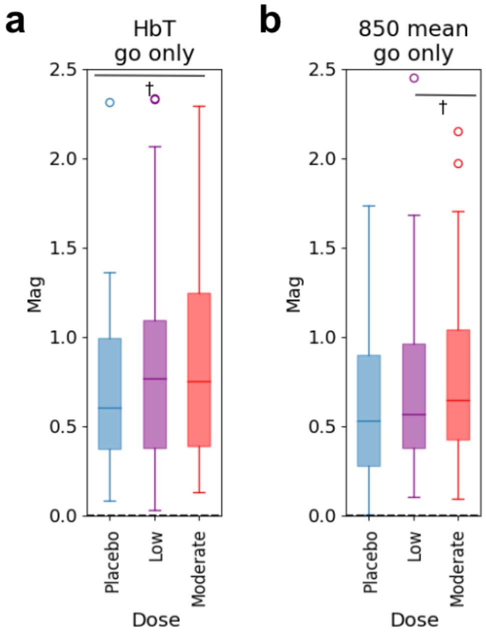

Supplementary Figure 5: Dose-dependent trends in magnitude metric during go-only condition of the Go/No-go Task.

a. The distributions of the HbT magnitude metric for go-only blocks across post-dose sessions (paired t-test: † trend towards significant difference between placebo and moderate dose: p=0.06). **b.** The distributions of the DToF mean moment (of 850nm) magnitude metric for go-only blocks across post-dose sessions (paired t-test: † trend towards significant difference between low and moderate dose: p=0.07).

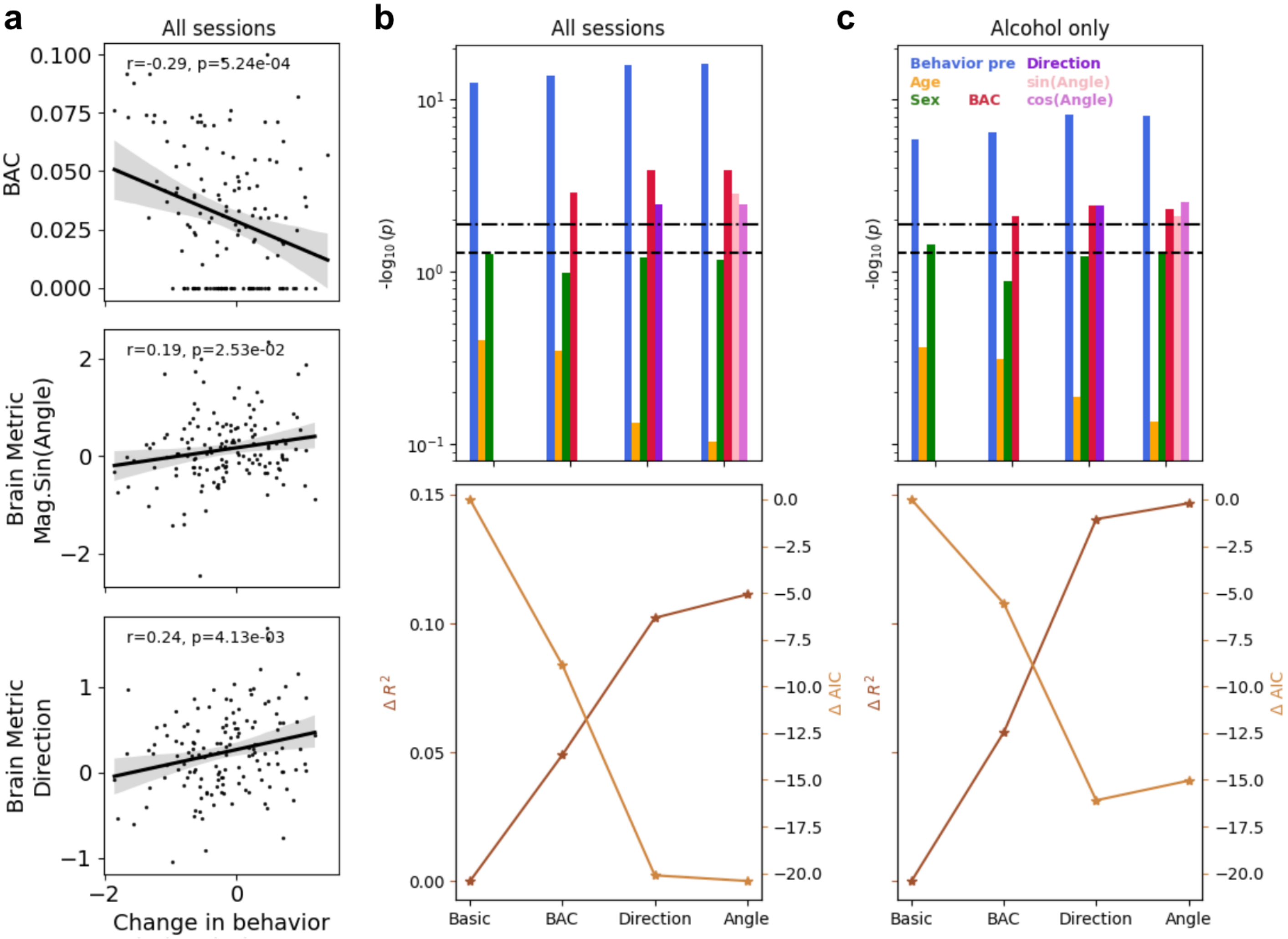

Supplementary Figure 6: DToF mean moment of 850nm lateralization metrics improved the prediction of behavioral performance in addition to measured BAC.

a. The change in behavioral performance as measured by post d-prime - pre d-prime was significantly correlated with BAC (top; pearson r=-0.29, p=5.24x10^{-4}), magnitude x sin(angle) (middle; pearson r=0.19, p=2.53x10^{-2}), and direction metric (bottom; pearson r=0.24, p=4.13x10^{-3}). **b.** Top) Statistical significance of the different factors in the four OLS models (four blocks shown on the x-axis) in predicting the post behavioral performance are reported as -log$_{10}$(p-value). We build off of the three factors in the basic model (pre behavioral performance: blue, age: yellow, sex: green), and include additional variables in separate OLS models: 1) BAC model included the direct measure of intoxication (BAC values, red) in addition to the basic model; 2) Direction model consisted of the direction metric (dark purple) in addition to the BAC model variables; 3) Angle model included the weighted sine and cosine of the angle metric (pink and light purple respectively) in addition to the BAC model variables. Note that both BAC values and lateralization metrics were significant factors in predicting post performance. Dashed lines indicate a significance level of 0.05 and 0.05/4 (after correcting for multiple comparisons using Bonferroni correction; Methods). Bottom) Overall OLS model performances are shown in terms of the change in R-squared values and the change in AIC (lower AIC values indicate better performance). **c.** Same as (b) but when only considering the sessions with alcohol consumption (i.e. low and moderate doses).

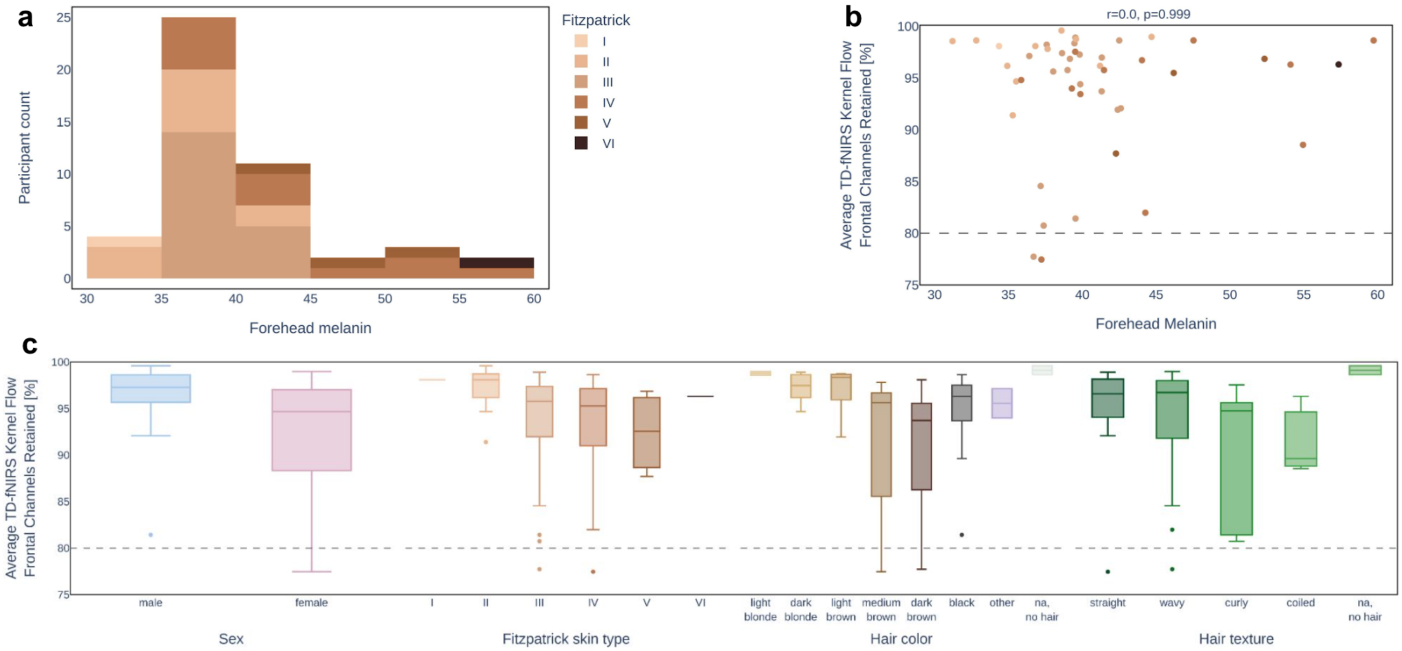

Supplementary Figure 7: The relationship between participants' skin color and the percentage of retained channels. **a.** A histogram of participants' forehead melanin levels (N=48), as measured by the colorimeter. Histogram is shaded by self-reported Fitzpatrick skin type (scale of I to VI, I being fairer and VI being darker skin). The participant pool contained a range of melanin levels. **b.** The percentage of retained prefrontal channels as a function of recorded forehead melanin levels. Each point represents a participant with the shade representing the participant's self-reported Fitzpatrick skin type (see a.). All but 2 participants had greater than 80% of channels retained (seen as the points above the dashed line), and all were above 75%. A lack of correlation indicated that the Flow1 system was able to perform well on a variety of skin colors. **c.** The distributions of retained prefrontal channels separated by various demographic factors (sex, Fitzpatrick skin type, hair color, and hair texture).

	Male (n=23)	Female (n=25)	All (n=48)
Age	32.48 ± 9.08	32.76 ± 10.59	32.63 ± 9.90
Mean drinks per week	6.24 ± 2.79	4.76 ± 2.45	5.43 ± 2.72

Supplementary Table 1: Participant Demographics and drinking habits.
